# 

**Published:** 1862-04

**Authors:** 


### CHICAGO MEDICAL JOURNAL.

**Terms, $2.00 per Annum.**

### CONTENTS OF THE APRIL NUMBER.

ORIGINAL COMMUNICATIONS.

PAGE.
Diphtheritic Conjunctivitis. By E. L. Holmes, M. D., of Chicago........ 189
Fort Donelson........................................................ 195
Case Illustrative of Prejudicial Influence of Quinine Improperly Administered. By Dr. Edward Lawrence, Hopper's Mills, Henderson County, Illinois............................................ 210
Two Cases in Practice. By J. B. Hoag, M. D., Windfall, Tipton Co., Ind., 212

SELECTED.

Clinical Lecture on Anæmia and Bloodletting. By Thomas K. Chambers, Lecturer on Systematic and Clinical Medicine.................. 216
Employment of Nitric Acid and Opium for the Treatment of Autumnal Forms of Diarrhœa and Dysentery......................... ... 226
Condition of the Mouth in Idiots. By J. Langdon H. Down, London, Physician to the Asylum for Idiots, Earlswood.................. 229
Pyrophosphate of Iron. By Jas. R. Nichols........................ 234

EDITORIAL AND MISCELLANEOUS........................................ 237

